# Comparative Antioxidant Protection of Cochlear Hair Cells from Ototoxins

**DOI:** 10.3390/molecules30183772

**Published:** 2025-09-17

**Authors:** Allen F. Ryan, Kwang Pak, Eun Jung Lee, Arwa Kurabi

**Affiliations:** 1 Department of Otolaryngology, University of California San Diego, La Jolla, CA 92093, USA; afryan@health.ucsd.edu (A.F.R.); kpak@health.ucsd.edu (K.P.); entejlee@jbnu.ac.kr (E.J.L.); 2 Department of Neurosciences, University of California San Diego, La Jolla, CA 92093, USA; 3 San Diego Veterans Administration Healthcare System, San Diego, CA 92161, USA; 4 Department of Otorhinolaryngology-Head & Neck Surgery, National University School of Medicine, Jeonju-si 54896, Jeonbuk, Republic of Korea

**Keywords:** inner ear, cisplatin, cochlear damage, hair cell protection

## Abstract

Many forms of damage to cochlear sensory cells involve reactive oxygen species (ROS). We previously screened 81 antioxidants in vitro for the ability to reduce cochlear hair cell (HC) damage by the ototoxic aminoglycoside gentamicin. Only 13 antioxidants produced significant reduction in HC loss, with the quinone antioxidants seratrodast and idebenone being most protective. Why so few antioxidants were protective is unclear, but most antioxidants have other properties that could enhance or detract from protection. In particular, seratrodast is a potent thromboxane A_2_ (TXA2) antagonist, while idebenone also strongly supports cell metabolism by enhancing mitochondrial function. We therefore asked whether a different TXA2 inhibitor (SQ-29548) or mitochondrial function enhancer (mitochonic acid) exhibited any HC protective ability in the same assay. In both cases, no significant protection from gentamicin was observed, indicating that the ROS scavenging activity of seratrodast and idebenone accounted for HC protection. Additionally, to assess the generality of HC protection by the two antioxidants, we assessed their potential for protection against cisplatin, an ototoxic anti-cancer drug that produces HC damage through a different mechanism than aminoglycosides, but which also involves ROS. High-dose seratrodast tested protected HCs from cisplatin damage, but not to the extent observed for gentamicin. High-dose idebenone was also protective, but even less than for seratrodast. Neither mitochonic acid nor SQ-29548 was protective against cisplatin. The results indicate that seratrodast and idebenone provide HC protection from gentamicin and cisplatin due to their free radical scavenging properties, but protection from cisplatin was less effective, presumably due to its different mechanism of ototoxicity.

## 1. Introduction

Ototoxicity is a significant side effect of several life-saving drugs, including aminoglycoside antibiotics and platinum-based chemotherapeutics. Aminoglycoside-induced ototoxicity may affect up to 50% of patients [1], while cisplatin or carboplatin can cause hearing loss in as many as 75–100% of patients [2,3]. Despite these risks, the clinical utility of these agents often makes their use unavoidable.

The primary cellular targets of ototoxic drugs are the sensory hair cells (HCs) of the inner ear, with outer hair cells (OHCs) exhibiting greater vulnerability than inner hair cells (IHCs) [4,5]. Although several intracellular pathways have been implicated in HC damage, including kinase activation [6], calcium release [7], DNA damage [8], autophagy [9], and apoptosis [6], reactive oxygen species (ROS) [10], are widely considered central contributors to acoustic injury. A gradient in HC glutathione content from base to apex of the cochlea [11], which inversely correlates with ototoxin sensitivity, further supports a role for redox imbalance. In animal models, antioxidant treatment or genetic upregulation of antioxidant pathways has been shown to delay or prevent ototoxic damage [3,10,12,13,14].

The success of experimental studies has engendered clinical trials of antioxidant treatment for ototoxic or noise-induced hearing loss. Kocyigit et al. [15] reported that N-acetyl cysteine (NAC) provided partial protection from amikacin ototoxicity at the highest frequencies tested. A trial of NAC in military trainees during firearms training also found a degree of hearing protection [16]. However, a NAC versus placebo trial prior to stapedectomy, in which drilling noise and surgical trauma can produce HC loss, showed equivalent levels of hearing loss (~10 dB), failing to demonstrate a protective effect [17]. Similarly, Kramer et al. [18] found that NAC treatment had no effect on temporary threshold shift induced by loud music, possibly due to reduced cochlear drug availability, variable trial designs, or differences in the molecular mechanism of damage.

Antioxidants can act via diverse mechanisms: scavenging ROS, disrupting lipid peroxidation chains, chelating metals, and upregulating endogenous enzymes [19], and their effects may vary with context [20]. Moreover, some antioxidants exhibit dose-dependent duality, protecting at low concentrations but promoting damage at higher levels [21], so comparative assessments in standardized models can be used to identify optimal antioxidants and an appropriate balance between protection.

**Figure 1:**
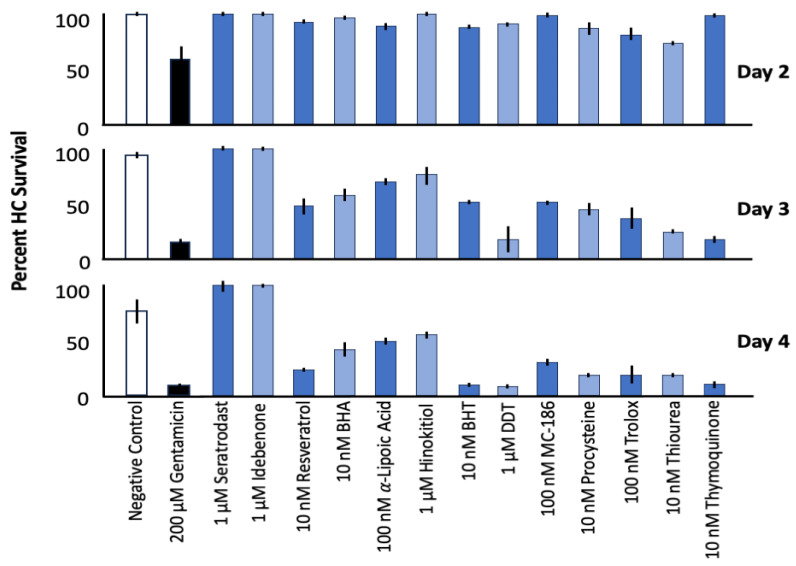
Maximum HC survival on Days 2 to 4 of gentamicin treatment with the 13/81 antioxidants screened in Ref. [22], which were effective protectants. The most effective dose of each antioxidant is presented, since protection varied by dose. This figure summarizes data presented by [22] in a different form.

To address this need, we previously conducted a high-content screen of 84 redox-active compounds (81 anti-antioxidants or inhibitors of free radical production and 3 pro-oxidants, listed in Supplementary Table S1) using an in vitro model of gentamicin-induced HC damage [22,23]. Only 13 of these compounds significantly improved HC survival as summarized in Figure 1, while six reduced it even in the absence of ototoxins, highlighting the importance of off-target effects in therapeutic development. Among the most effective compounds identified were idebenone and seratrodast, two quinone-based compounds with additional biological activities.

Idebenone is a short-chain benzoquinone derivative originally developed as a synthetic analog of Coenzyme Q_10_ (ubiquinone), a key component of the mitochondrial electron transport chain, with superior lipophilicity and cellular uptake [24]. Its reported mechanisms of action include the inhibition of peroxide formation, scavenging of free radicals and neutralization of ROS. However, in cell-free systems Idebenone has not demonstrated potent antioxidant activity, as would be expected from its structure. In cellular systems, conversion of Idebenone to its hydroxyquinone form is thought to underlie its antioxidant effects [25,26]. It has shown potent antioxidant and mitochondrial-protective effects in multiple biological models of oxidative stress-mediated injury, including gentamicin-induced ototoxicity [27], and noise-induced cochlear damage [28], where it reduced reactive oxygen species formation, preserved mitochondrial membrane potential, and protected hair cell viability in vitro and in vivo, respectively.

Seratrodast is structurally related to leukotrienes. While not an antioxidant itself, it becomes a potent radical-trapping agent upon reduction to its hydroxyquinone form (as is analogous to ubiquinone and vitamin K) [29]. Seratrodast is also a potent thromboxane A_2_ receptor (TP) antagonist [30], clinically approved for the treatment of asthma. It exerts anti-inflammatory and vasodilatory effects by blocking thromboxane-mediated platelet aggregation and microvascular constriction [31], mechanisms implicated in cochlear ischemia and oxidative injury [32].

The current study was designed to explore whether the benefits of idebenone and seratrodast might be attributed to their secondary mechanisms, mitochondrial enhancement and thromboxane inhibition, respectively. To achieve this, mitochonic acid, a small molecule derived from indole-3-acetic acid [33], and SQ-29548, a thromboxane A_2_ receptor antagonist with anti-inflammatory activity [34], were used as mechanistic probes (Table 1). Mitochonic acid is known to regulate mitochondrial energy metabolism by stabilizing mitochondrial membrane potential, enhancing ATP production, and reducing mitochondrial reactive oxygen species (ROS) levels through mechanisms independent of electron transport chain complexes I–IV [35]. SQ-29548, on the other hand, inhibits thromboxane A_2_ receptor–mediated signaling implicated in vasoconstriction, platelet aggregation, and inflammatory responses [36], thereby serving as a pharmacological tool to dissect the contribution of the thromboxane pathway to cochlear injury. However, neither mitochonic acid nor SQ-29548 has any reported antioxidant properties. Furthermore, we assessed the protective capacity of idebenone and seratrodast against cisplatin-induced ototoxicity. This approach was designed to evaluate the mechanism-specific and general HC protective potential of the four compounds.

## 2. Materials and Methods

### 2.1. Animals

Transgenic mice in which eGFP is selectively expressed in HCs under the control of a *pou4f3* promoter construct [37] were used for all experiments. This study was performed to National Institutes of Health (NIH) guidelines and was approved by the Institutional Animal Care and Use Committee of the San Diego VA Medical Center. 

### 2.2. Explant Preparation

The organ of Corti was micro-dissected from the cochleae of 3–5 day old *pou4f3*/*eGFP* mouse pups. The apical region of the epithelium, which is much less sensitive to ototoxicity, was discarded. The basal and middle turns of the organ were divided with a diamond scalpel into micro-explants, each consisting of ~20 inner HCs and the 60 associated outer HCs. The explants were plated into flat-bottom 96-well plates (Corning^®^, Corning, NY, USA), in media consisting of DMEM:F-12 (Gibco, Thermo Fisher Scientific, Waltham, MA, USA) plus Penicillin (Gibco) and 5% FBS (Gibco), with antibiotic kept below the threshold for HC damage as determined by comparing culture with and without Penicillin. The day of plating was termed Day 0.

### 2.3. Compound Testing

Control explants were maintained in media alone for 24 h, while each experimental explant was pre-treated with a potentially protective compound over the same period, with one of the various test compounds in culture media. Seratrodast (Selleckchem, Houston, TX, USA) and idebenone (Selleckchem, Houston, TX, USA) were tested at concentrations of 10, 100, and 1000 nM in media, based on our prior study [22]. Mitochonic acid 5 (Cayman, Ann Arbor, MI, USA) was used at the concentrations of 1, 2.5, 5, and 10 µM, and SQ-29548 (Cayman, Ann Arbor, MI, USA) was at the concentrations of 50, 100, 500, and 800 nM in media, bracketing published EC_50_s provided by the manufacturer Cayman chemicals. All cultures were performed with 0.1% DMSO (Sigma-Aldrich, Saint Louis, MO, USA). After the initial 24 h, the explants were imaged using fluorescence microscopy. This time point was termed Day 1. All explants were then maintained for an additional three days, termed Days 2, 3, and 4. Each condition was performed in triplicate. Negative controls were maintained in media alone (no treatment), and positive controls were in media containing 200 µM gentamicin (stock at 20 mM, Sigma-Aldrich, Saint Louis, MO, USA), or 30 µM cisplatin (stock at 3 mM, Sigma-Aldrich, Saint Louis, MO, USA). These concentrations were selected because they consistently produced significant hair cell loss by Day 1 and through Day 3 in culture, providing a reproducible level of injury that allowed us to test protective effects under standardized conditions. Experimental explants were maintained with a test compound at the above concentrations plus the ototoxin. An additional control consisted of the highest concentration of the test compound without an ototoxin challenge. All experiments were conducted with three explants and then replicated (*N* = 6). The performance of experiments on different days, and with different media and ototoxin batches, required verification that both control conditions were consistent plate-to-plate, and thus both negative and positive controls were included in each 96-well plate.

All chemicals used in this study were of the highest purity from the supplier (≥99%, HPLC grade) and, when applicable, were supplied with cell culture–validated certification from the manufacturer to ensure suitability for in vitro experiments.

GFP-positive cells were imaged by fluorescence microscopy at the end of Day 1 and at the end of Days 2, 3, and 4 of treatment. Any micro-explants that did not attach and flatten in the well by Day 1 were excluded from the assay, since HC counts could not be accurately quantified at that time. HC counts, including both inner and outer HCs together, were performed in ImageJ (1.x, NIH, Bethesda, MD, USA) and were normalized to the number of HCs present on Day 1, at the start of ototoxin treatment. HC survival curves were generated for controls and for each experimental compound and dosage. The data were converted to percent of HCs on day 1.

### 2.4. Statistical Analysis

Statistical analysis for each condition utilized individual explant counts and was performed using GraphPad Prism 6 and StatView 5. The Kruskal–Wallis nonparametric ANOVA was used to detect treatment effects (df = 6). Individual condition comparisons were performed with the nonparametric Mann–Whitney U test, with Bonferroni correction for multiple comparisons. For Figure 1 and the tables, standard errors of the mean were calculated from the non-normalized HC counts.

## 3. Results

### 3.1. Control Explants

To evaluate ototoxic effects, we quantified hair cell survival following exposure to cisplatin or gentamicin. Both agents induced time-dependent HC loss compared to untreated (negative) controls. The cochlear explants maintained in standard culture conditions demonstrated high levels HC survival throughout the 4-day observation period, with minimal cell loss and no detectable morphological abnormalities.

**Figure 2:**
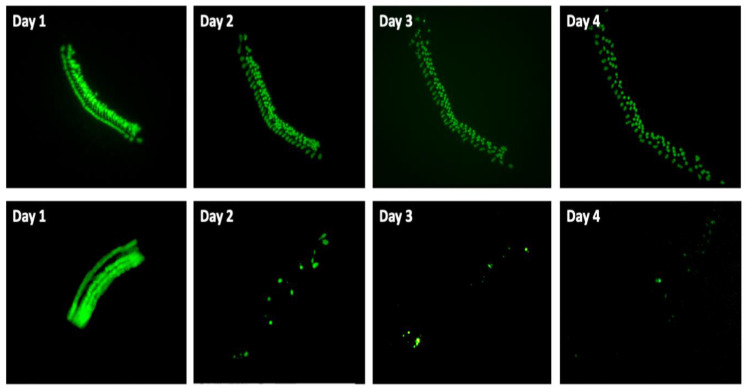
Culture of control micro-explants from the neonatal Pou4f3/GFP mouse used in this study. The **upper row** illustrates culture of a negative control (untreated) explant, in which slight HC loss occurred after 1 or 2 days of culture without gentamicin exposure, with modest losses on day 3. The **lower row** presents a positive control explant treated with gentamicin beginning on Day 1. Massive HC losses are observed by Day 2, and almost all cells are lost by Day 4.

Negative (untreated) controls in the gentamicin experiments showed an average of 96% HC survival on Day 2, and 93% on Day 3 (93%), but by Day 4, survival was reduced to 81%, significantly lower than on Day 1. Representative images of GFP-positive HCs in negative control wells and in gentamicin-treated wells are presented in Figure 2. HC counts from negative and positive control explants in each plate were generated, converted to percent survival relative to Day 1 (D1, just prior to gentamicin/cisplatin exposure), and averaged across all plates and replicates. Positive gentamicin control explants showed 25% survival on Day 2, 7% survival on Day 3, and 4% survival on Day 4. Negative (untreated) control explants in cisplatin plates exhibited 93% HC survival on Day 2, 90% HC survival on Day 3, and 84% HC survival on Day 4. Cisplatin positive controls showed 67% survival on Day 2, 59% on Day 3, and 39% on Day 4.

**Figure 3:**
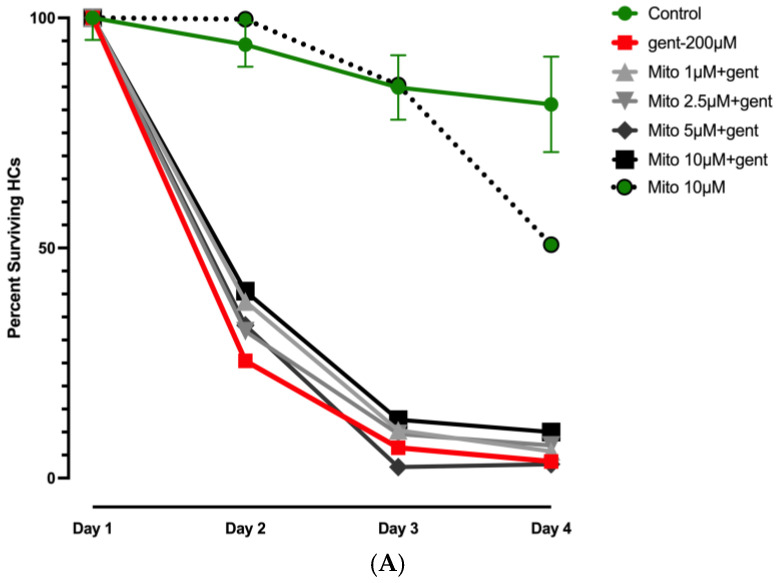

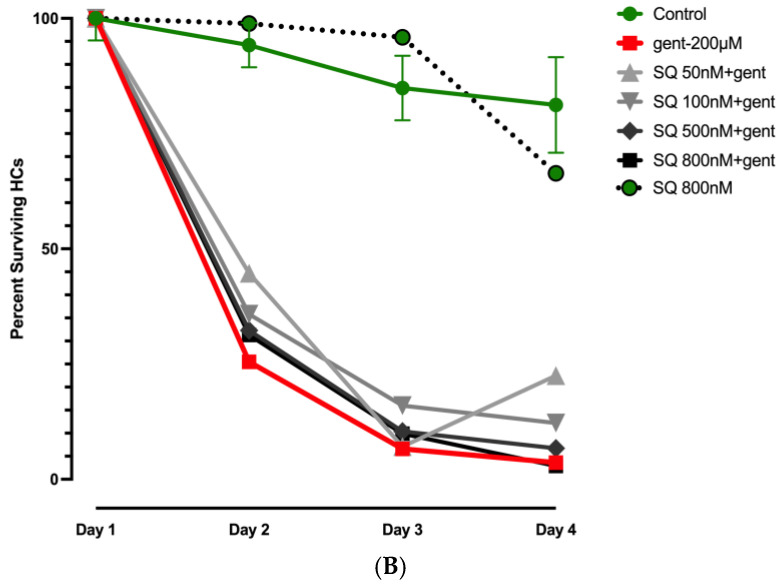
Normalized Day 1–4 survival curves of micro-explant HCs treated with gentamicin (gent) plus an alternative process compound. (**A**). Effects of four dosages of the mitochondrial metabolic enhancer mitochonic acid (Mito) on HC loss during treatment with gentamicin. No effect on gentamicin-induced HC loss was noted at any dosage. (**B**). Similarly, no protective effect of the anti-inflammatory prostaglandin and thromboxane inhibitor SQ-29548 (SQ) was observed.

**Figure 4:**
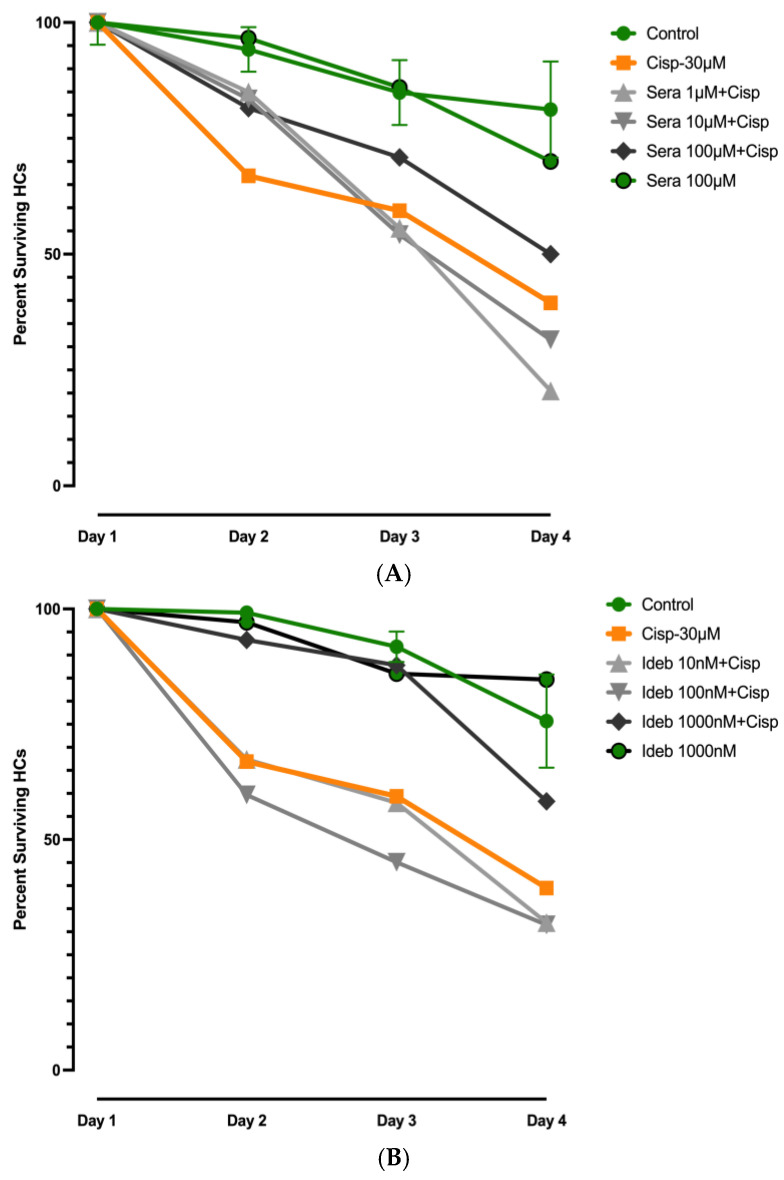
Normalized Days 1–4 survival curves of micro-explant HCs treated with 30 µM cisplatin (Cisp). (**A**). Effect of four dosages of seratrodast on ototoxicity induced by cisplatin damage. Significant protection of HCs was observed for all dosages at Day 2. On Day 3 and Day 4, only the highest dosage (100 µm) was protective. (**B**). Effect of three dosages of idebenone on cisplatin ototoxicity. Protection was observed on Day 2 and Day 3 at 1000 nM dose.

**Figure 5:**
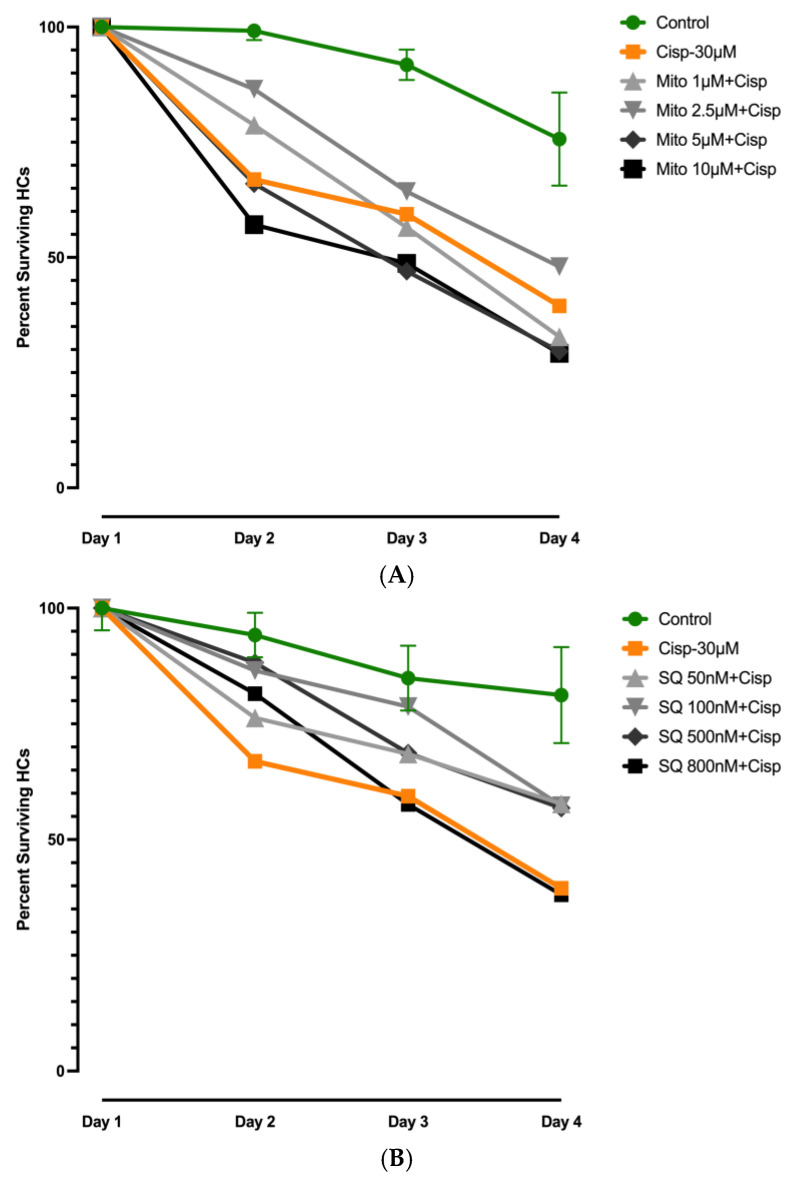
Effects of mitochonic acid (Mito) and SQ-29548 on cisplatin-induced hair cell (HC) loss. (**A**). Percent HC survival over four days in cochlear explants treated with cisplatin alone or in combination with mitochonic acid at four dosages. Mitochonic acid did not confer significant protection at any concentration. (**B**). Effect of four dosages of SQ-29548 on cisplatin-induced ototoxicity. A slight elevation in HC survival was observed at 100 nM on Days 2–3, but the effect was not statistically significant and declined by Day 4.

Control results for gentamicin are illustrated in Figure 3 and Supplementary Table S2, while those for cisplatin are presented in Figure 4 and Figure 5 and Supplementary Tables S3 and S4. All positive control counts on day 2–4 were significantly different from those on Day 1 for both ototoxins (Supplementary Tables S2–S4). As expected, Kruskal–Wallis nonparametric ANOVA and Mann–Whitney post hoc tests showed significant differences between negative and positive controls for both ototoxins from Day 2 to Day 4 (*p* < 0.0001).

### 3.2. Effects of Mitochonic Acid or SQ-29548 on Gentamicin-Induced HC Loss

Figure 3A illustrates the response of HC to mitochonic acid (Mito) treatment in the context of gentamicin-induced ototoxicity. It is clear from the figure that mitochonic acid had no significant protective effect at any dosage. Comparison of these results with our previously published data on idebenone treatment of gentamicin (gent) ototoxicity [22] indicated highly statistically significant differences (*p* < 0.001) between mitochonic acid and idebenone effects on gentamicin ototoxicity at 100 nM and 1000 nM. Conversely, at the highest concentration (10 µM), mitochonic acid produced a HC count statistically lower than that of the negative control on Day 4 (*p* < 0.001), indicating delayed toxicity effects.

As illustrated in Figure 3B, SQ-29548 acid at the lowest dosage employed (50 nM) showed slightly but significantly (*p* < 0.02) higher HC survival against gentamicin on Days 2, 3, and 4, indicating a protective effect. Notably, no significant protection was noted at the longer gentamicin exposures with the higher concentrations. Comparison of these results with our previously published data on seratrodast treatment during gentamicin ototoxicity assay [22] showed highly statistically significant differences (*p* < 0.001) between the effects of SQ-29548 and those of seratrodast applied at 0.1, 1, or 10 µM.

### 3.3. Effects of Seratrodast or Idebenone on Cisplatin Ototoxicity

Figure 4A illustrates the effects of seratrodast on cisplatin-induced ototoxicity in a dose and time-dependent manner. On Day 2, all dosages of seratrodast significantly reduced HC loss (*p* ≤ 0.02). The highest dosage, 100 µM, reduced HC loss but did not reach significance (*p* = 0.051) on Day 3, but the reduction in HC loss was significant on Day 4 (*p* < 0.01), indicating sustained protective efficacy at this concentration. Notably, 100 μM seratrodast alone did not induce any detectable toxicity alone, confirming its safety in this explant model.

Figure 4B presents the effects of idebenone on cisplatin (cisp) ototoxicity. At the highest concentration tested (1000 nM), idebenone provided significant protection on Days 2 and 3 (*p* < 0.05), but the effect was no longer significant by Day 4. Lower concentrations of idebenone did not produce a protective effect at any time point.

### 3.4. Effects of Mitochonic Acid or SQ-29548 on Cisplatin-Induced HC Loss

Figure 5 illustrates the effects of mitochonic acid and SQ-29548 on cisplatin-induced hair cell loss. Treatment with Mito at 100 nM or 1000 nM did not significantly alter the course of HC loss compared to cisplatin alone, indicating no protective effect at these concentrations. Similarly, SQ-29548 provided no meaningful protection against cisplatin-induced damage over the time course of the assay. While the 1 μM dose showed a slight improvement on Days 2 and 3, this effect was not sustained or statistically significant, and no dose provided durable protection through Day 4. These results indicate that neither mitochonic acid nor SQ-29548 confers consistent or effective protection against cisplatin ototoxicity in this model.

Detailed data, including SEMs, are presented in Supplementary Tables S2–S4.

## 4. Discussion

In summary, we found that compounds blocking non-antioxidant activities of seratrodast (TBXA receptor inhibition) or idebenone (enhancement of mitochondrial metabolism) did not mimic their protective activities against gentamicin ototoxicity. Specifically, the lack of protection observed with mitochonic acid, a mitochondrial enhancer, and SQ-29548, a thromboxane A_2_ receptor antagonist, indicates that idebenone and seratrodast likely engage pathways beyond mitochondrial stabilization or thromboxane blockade, most likely the antioxidant properties of their hydroxyquinone derivatives. In addition, we noted modest protective effects for both idebenone and seratrodast against cisplatin-induced HC damage. However, this protection was less effective than for gentamicin, even though the degree of HC damage for cisplatin-only explants was generally less than for those treated with gentamicin.

Our prior screen of 81 anti-oxidant compounds [22,23] found that only 13 were able to protect against high-dose gentamicin (Figure 1). Effective compounds included several antioxidant classes, including quinone antioxidants, phenolic antioxidants, a sulfur-containing antioxidant, a glutathione precursor, a vitamin E analog, and a tropolone-derived iron chelator. The most effective classes were quinone antioxidants and phenolic antioxidants. While this indicates that many modes of antioxidant activity are protective, it does not include the fact that many antioxidants have alternative activities that could affect ototoxicity. This includes the quinone antioxidants seratrodast and idebenone, the most effective protectants in our screen. Seratrodast inhibition of TBXA was of interest given that lipid peroxidation induces TXA expression [38,39]. However, inhibition of the TXA2 receptor did not have a protective effect. Our results with mitochonic acid similarly indicate that the metabolic effects of idebenone are not responsible for HC protection. Rather, it is the radical scavenging activity of seratrodast and idebenone that underlies HC protection.

This conclusion is supported by the comparison of the 13 protective antioxidants in our original screen [22]. The four most effective compounds are primarily free radical scavengers. Fewer compounds with other antioxidant effects, including enhancement of endogenous antioxidants, metal chelation, reduction in lipid peroxidation reactions, or anti-inflammatory properties, were effective. This result suggests that free radical scavenging may be the most important antioxidant mechanism protecting HCs from gentamicin. However, other unexplored pleiotropic mechanisms of idebenone and seratrodast, beyond mitochondrial stabilization or thromboxane blockade, may be essential for their high level of efficiency in protection against gentamicin-induced hair cell damage, compared to other antioxidants. To further explore such alternative mechanisms, follow-up studies using, for example, apoptosis, ferroptosis or JNK inhibitors in the same assay setting, could determine whether additional cell death mechanisms that contribute to hair cell injury and protection are inhibited by these two compounds.

The issue of whether either seratrodast or idebenone might protect HCs from cisplatin damage hinges upon the difference in the mechanism of damage between aminoglycosides and platinum-based antineoplastic agents. Aminoglycosides have been shown to increase calcium entry into HC mitochondria, which in turn increases ROS production and release through the mitochondrial membrane [40]. Cisplatin accumulates in the mitochondria, leading to their damage and the release of ROS [3,41,42]. One might thus expect similar levels of protection for the two ototoxins. However, cisplatin is also well known to bind to DNA and block translation and replication [43]. This can lead to cell death and is the major mechanism of its anticancer effectiveness [44]. Moreover, cisplatin has been shown to damage DNA in HCs [45]. The existence of this alternative mechanism of HC damage may explain why idebenone and seratrodast were significantly less effective in protection against cisplatin than against gentamicin. A similar finding was reported by [46], who found that the SOD dismutase mimetic M40403 protected against gentamicin, but not cisplatin, ototoxicity. Unfortunately, this compound was not included in our prior REDOX library study. The addition of DNA damage presumably renders cisplatin ototoxicity less sensitive to these two antioxidants than gentamicin-induced HC damage. In the future, it would be illuminating to test the entire REDOX library against cisplatin ototoxicity, to determine if other compounds might be more effective than seratrodast or idebenone.

Treatment with mitochonic acid or SQ-29548 had no effect on cisplatin ototoxicity, indicating that neither metabolic enhancement nor inhibition of TXA2 receptor activation significantly altered the mechanisms of cisplatin HC damage. This is despite evidence for HC mitochondrial damage [47] by this ototoxin, and the role of TXA in cisplatin-induced renal tubule damage [48]. The lack of protection observed here suggests that cisplatin ototoxicity likely involves additional or alternative mechanisms beyond mitochondrial dysfunction or thromboxane signaling, warranting further studies using inhibitors of apoptosis, ferroptosis, or stress kinase pathways as noted above to better define the molecular basis of hair cell injury in this model.

Several other studies have demonstrated the antioxidant activity of seratrodast and idebenone in in vitro cellular systems [49,50,51]. For example, Suno and Nagaoka [52] observed that idebenone treatment reduced lipid peroxidation in the mitochondria of brain cells by 45–54%, while Miyamoto et al. [53] noted the reduction in glutamate-induced ROS in a neuronal cell line to control levels. Hao et al., [54] found that seratrodast treatment of HT22 cells reduced ROS levels induced by erastin, which reduces glutathione levels, by 80%. These studies provide evidence for the antioxidant activity of idebenone and seratrodast; however, they did not investigate their additional activities. The results of the present investigation provide evidence that the antioxidant properties of these two compounds are responsible for robust HC protection against gentamicin ototoxicity. Why they are superior to other antioxidants, even those with similar mechanisms of action, remains to be determined.

There are clear limitations to our study. The use of neonatal HCs does not reflect the state of human HCs typically exposed to ototoxins, which even in children function at the adult level. We used immature HCs since adult mammalian HCs do not survive well in culture. However, their responses to toxins, antioxidants, mitochonic acid, or SQ-29548 may differ significantly from those of adult HCs. Moreover, our in vitro study does not address bioavailability to HCs in vivo. We also used a very high dose of gentamicin, expecting it to destroy nearly all HCs, since the original study of Noack et al. [22] was designed to identify only strong HC protectants. A lower dose may have revealed more protective antioxidants in addition to the 13 identified by Noack et al. [22] In contrast, we used a dose of cisplatin that was expected to destroy approximately 50% of HCs, as our goal was to identify any protective effect of seratrodast or idebenone. A more severe dose of cisplatin may have overwhelmed their modest protective effects.

Our findings highlight the value of both natural and synthetic quinone-based compounds in the search for therapeutics that protect against ototoxic injury. Although idebenone and seratrodast are synthetic, their structures are derived from naturally occurring molecules, ubiquinone and leukotrienes, respectively—illustrating how synthetic analogs can be strategically designed to enhance or expand the biological functions of natural compounds. Their limited efficacy against cisplatin-induced damage, despite the lower baseline toxicity used, underscores the complexity of ototoxic mechanisms and the need for context-specific protective strategies. However, the results support the potential of leveraging both natural and rationally designed synthetic compounds in the eventual development of targeted therapies for hearing preservation.

## Figures and Tables

**Table 1:** Chemical Structure of Seratrodast, SQ-29548, Idebenone and Mitochonic acid.

Name	Abbreviation	Structure	Chemical Formula Molecular Weight (g/mol)	Class
Seratrodast	Sera	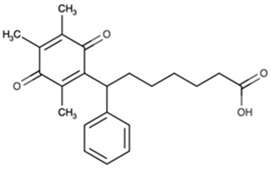	C_22_H_26_O_4_ (354.45)	Synthetic quinone TXA2 receptor antagonist
SQ-29548	SQ	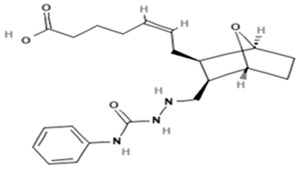	C_21_H_29_N_3_O_4_ (387.47)	Synthetic TXA2 receptor antagonist
Idebenone	Ideb	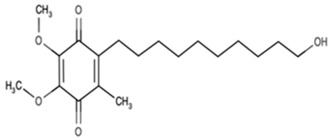	C_19_H_30_O_5_ (338.44)	Synthetic ubiquinone analog
Mitochonic Acid 5	Mito	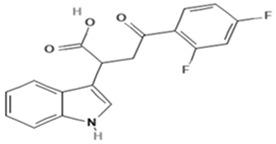	C_19_H_13_F_2_NO_4_ (329.30)	Mitochondrial targeted metabolic modulator

## Data Availability

The original contributions presented in this study are included in the article/Supplementary Material. Further inquiries can be directed to the corresponding author.

## Supplementary Materials

The following supporting information can be downloaded at: https://www.mdpi.com/article/10.3390/molecules30183772/s1, Table S1. List of Inhibitors in the Redox Library. Table S2. Gentamicin experiments (Figure 3). Table S3. Cisplatin Idebenone and Seratrodast Experiments (Figure 4). Table S4. Cisplatin Mitochonic Acid and SQ-29548 Experiments (Figure 5).
