# Supplementary material for: Comparative Antioxidant Protection of Cochlear Hair Cells from Ototoxins
Source: Molecules. 2025 Sep 17;30(18):3772. doi: 10.3390/molecules30183772 (PMC12473111; doi:10.3390/molecules30183772)
Supplement: Supplementary file 1 [file molecules-30-03772-s001.zip › Supplementary Table S1.pdf]

Supplementary Table S1: List of Inhibitors in the Redox Library

The Screen-Well® Redox Library (Enzo Life Sciences, Farmingdale, NY, USA) was used as the source for all compounds listed.

No.	Compound	Activity	Origin
1	Promethazine·HCl	Secondary anti-oxidant	Synthetic
2	Cumene hydroperoxide	Aryl hydroperoxide	Synthetic
3	β-Lapachone	Undergoes futile redox cycles	Synthetic
4	Resveratrol	Stilbene phenolic antioxidant	Natural
5	Hydroquinone	Phenolic antioxidant	Synthetic
6	TEMPOL	SOD mimetic	Synthetic
7	Ferulic acid ethylester	Phenolic antioxidant	Natural
8	D-α-Tocopherylquinone	Oxidized vitamin E	Synthetic
9	Seratrodast	Quinone antioxidant	Synthetic
10	Idebenone	Quinone antioxidant	Synthetic
11	tert-Butylhydroquinone	Phenolic antioxidant	Synthetic
12	HBED·HCl·H2O	Metal chelator	Synthetic
13	Ambroxol	Nonphenolic antioxidant	Synthetic
14	L-Ergothioneine	Endogenous antioxidant	Natural
15	Hinokitiol	Metal chelator	Synthetic
16	Epigallocatechin gallate	Polyphenol	Natural
17	Procysteine	Glutathione precursor	Synthetic
18	Trolox	Short-chain vitamin E analog	Synthetic
19	MCI-186	Nonphenolic antioxidant	Synthetic
20	U83836E·2HCl	Antioxidant Lazaroid	Synthetic
21	U74389G maleate	Antioxidant Lazaroid	Synthetic
22	GERI-BP002A	Phenolic antioxidant	Synthetic
23	Apigenin	Flavone antioxidant	Natural
24	Terbinafine·HCl	Free radical quencher	Synthetic
25	Rosmarinic acid	Phenolic antioxidant	Natural
26	Piceatannol	Stilbene phenolic antioxidant	Natural
27	AA-861	Quinone antioxidant	Synthetic
28	CDC	Phenolic antioxidant	Synthetic
29	Ebselen	Glutathione peroxidase mimetic	Synthetic
30	Genistein	Isoflavone antioxidant	Natural
31	Curcumin	Phenolic antioxidant	Natural
32	Phenidone	Nonphenolic antioxidant	Synthetic
33	Gossypol	Phenolic antioxidant	Synthetic
34	Gentisic acid	Phenolic antioxidant	Synthetic
35	Caffeic acid	Phenolic antioxidant	Natural
36	Baicalein	Flavone antioxidant	Natural
37	Esculetin	Coumarin antioxidant	Natural
38	N-Propyl gallate	Phenolic antioxidant	Synthetic
39	ETYA	Acetylenic antioxidant	Synthetic
40	CAPE	Phenolic antioxidant	Synthetic
41	NDGA	Phenolic antioxidant	Natural
42	Capsaicin	Phenolic antioxidant	Natural
43	BHT	Phenolic antioxidant	Synthetic
44	BHA	Phenolic antioxidant	Synthetic
45	Bakuchiol	Phenolic antioxidant	Synthetic
46	DL-α-Lipoic acid	Sulfur-containing antioxidant	Synthetic

47	Eugenol	Phenolic antioxidant	Natural
48	Melatonin	Nonphenolic antioxidant	Synthetic
49	N-Acetyl-Cysteine	Thiol-containing reducing agent	Synthetic
50	D-γ-Tocopherol	Phenolic antioxidant	Synthetic
51	Tocopherol succinate	Phenolic antioxidant	Synthetic
52	Ascorbic acid	Ascorbate-type antioxidant	Synthetic
53	Ascorbyl palmitate	Lipohilic ascorbate	Synthetic
54	n-Octyl caffeate	Phenolic antioxidant	Synthetic
55	Paeonol	Phenolic antioxidant	Synthetic
56	Protocatechuic acid	Phenolic antioxidant	Synthetic
57	Glutathione	Thiol-containing reducing agent	Natural
58	Carvedilol	Nonphenolic antioxidant	Synthetic
59	Diludin	Dihydropyridine-type antioxidant	Synthetic
60	Carnosic acid	Phenolic antioxidant	Natural
61	Tanshinone IIA	1,2-Quinone antioxidant	Natural
62	Probucol	Phenolic antioxidant	Synthetic
63	EPA	Polyunsaturated radical scavenger	Natural
64	DCHA	Polyunsaturated radical scavenger	Natural
65	bis-demethoxycurcumin	Phenolic antioxidant	Synthetic
66	Ibuproxam	Metal chelator	Synthetic
67	Ciclopirox ethanolamine	Hydroxyl radical scavenger	Synthetic
68	Thymoquinone	Quinone antioxidant	Natural
69	Thiourea	Thiol-containing reducing agent	Synthetic
70	DTT	Thiol-containing reducing agent	Synthetic
71	N-Ethylmaleimide	Thiol trap	Synthetic
72	Buthionine sulfoximine	Glutathione biosynthesis inhibitor	Synthetic
73	Anethole trithione	Sulfur-containing antioxidant	Natural
74	TEMPO	Nitroxyl radical	Synthetic
75	D609	Antioxidant	Synthetic
76	Captopril	Thiol-containing reducing agent	Synthetic
77	Disulfiram	Sulfur-containing antioxidant	Synthetic
78	1,2-Dithiole-3-thione	Sulfur-containing antioxidant	Synthetic
79	Selenomethionine	Selenium-containing antioxidant	Synthetic
80	Tetramethylpyrazine	Nonphenolic antioxidant	Synthetic
81	Ethoxyquin	Nonphenolic antioxidant	Synthetic
82	Canthaxanthin	Polyunsaturated radical scavenger	Natural
83	β-carotene	Polyunsaturated radical scavenger	Natural
84	Retinyl palmitate	Polyunsaturated radical scavenger	Natural
