# Supplementary material for: Comparative Antioxidant Protection of Cochlear Hair Cells from Ototoxins
Source: Molecules. 2025 Sep 17;30(18):3772. doi: 10.3390/molecules30183772 (PMC12473111; doi:10.3390/molecules30183772)
Supplement: Supplementary file 1 [file molecules-30-03772-s001.zip › Supplementary Tables S2-S4.pdf]

Supplementary Table S2. Gentamicin experiments (Figure 3)

**Day 2**	HCs Mean	Std error	P value vs Gent	
Control	94.2	4.8	<0.0001	S
Gent 200 µM	25.5	3.2	--------	
Gent + Mito 1 µM	38.4	6.2	0.099	
Gent + Mito 2.5 µM	31.9	8.3	0.400	
Gent + Mito 5 µM	33.2	2.6	0.315	
Gent + Mito 10 µM	40.6	7.3	0.056	
Mito 10 µM	99.7	0.2	<0.0001	S
Gent + SQ 50 nM	44.7	2.7	0.018	S
Gent + SQ 100 nM	35.8	6.3	0.183	
Gent + SQ 500 nM	32.3	4.7	0.376	
Gent + SQ 800 nM	31.3	8.2	0.449	
SQ 800 nM	98.9	0.0	<0.0001	S

**Day 3**	HCs Mean	Std error	P value vs Gent	
Control	84.9	7.0	<0.0001	S
Gent 200 µM	6.6	1.0	--------	
Gent + Mito 1 µM	10.4	2.8	0.594	
Gent + Mito 2.5 µM	9.6	5.5	0.676	
Gent + Mito 5 µM	2.4	1.4	0.558	
Gent + Mito 10 µM	12.7	3.9	0.394	
Mito 10 µM	85.4	4.1	<0.0001	S
Gent + SQ 50 nM	7.1	0.2	0.295	
Gent + SQ 100 nM	16.0	5.5	0.194	
Gent + SQ 500 nM	10.4	2.3	0.594	
Gent + SQ 800 nM	9.9	5.7	0.642	
SQ 800 nM	95.9	5.4	<0.0001	S

**Day 4**	HCs Mean	Std error	P value vs Gent	
Control	81.2	10.4	<0.0001	S
Gent 200 µM	3.6	1.0	--------	
Gent + Mito 1 µM	5.8	2.8	0.758	
Gent + Mito 2.5 µM	7.1	5.5	0.633	
Gent + Mito 5 µM	3.0	1.4	0.941	
Gent + Mito 10 µM	10.0	3.9	0.386	
Mito 10 µM	50.7	4.1	<0.0001	S

Gent + SQ 50 nM	22.5	0.8	0.016	
Gent + SQ 100 nM	12.2	4.6	0.250	
Gent + SQ 500 nM	6.7	2.8	0.671	
Gent + SQ 800 nM	2.9	1.0	0.930	
SQ 800 nM	66.4	10.4	<0.0001	S

Supplementary Table S3. Cisplatin Idebenone and Seratrodast Experiments (Figure 4)

**Day 2**	HCs Mean	Std error	P value vs Cis	
Control	99.2	1.4	0.015	S
Cisplatin 30 µM	66.9	9.7	--------	
Cis + Ideb 10 nM	67.4	5.6	0.967	
Cis + Ideb 100 nM	59.7	7.1	0.567	
Cis + Ideb 1000 nM	93.3	5.7	0.043	S
Ideb 1000 nM	97.1	1.8	0.022	S
Cis + Sera 1 µM	84.6	2.8	0.012	S
Cis + Sera 10 µM	83.5	5.5	0.014	S
Cis + Sera 100 µM	81.5	11.7	0.020	S
Sera 100 µM	96.6	1.7	0.002	S

**Day 3**	HCs Mean	Std error	P value vs Cis	
Control	91.9	3.5	0.004	S
Cisplatin 30 µM	36.8	11.0	--------	
Cis + Ideb 10 nM	58.0	9.3	0.912	
Cis + Ideb 100 nM	45.1	6.8	0.264	
Cis + Ideb 1000 nM	87.8	7.4	0.030	S
Ideb 1000 nM	86.0	4.2	0.042	S
Cis + Sera 1 µM	55.6	4.41	0.258	
Cis + Sera 10 µM	54.2	15.3	0.295	
Cis + Sera 100 µM	70.9	16.2	0.051	
Sera 100 µM	85.7	8.2	0.008	S

**Day 4**	HCs Mean	Std error	P value vs Cis	
Control	75.7	10.1	0.018	S
Cisplatin 30 µM	39.5	85.3	--------	
Cis + Ideb 10 nM	32.0	4.1	0.608	
Cis + Ideb 100 nM	31.5	1.6	0.583	
Cis + Ideb 1000 nM	58.3	5.3	0.204	
Ideb 1000 nM	84.7	3.1	0.004	S
Cis + Sera 1 µM	20.5	3.96	0.317	
Cis + Sera 10 µM	31.6	9.92	0.078	
Cis + Sera 100 µM	49.7	12.0	0.005	S
Sera 100 µM	70.0	14.2	0.002	S

Supplementary Table S4. Cisplatin Mitochonic Acid and SQ-29548 Experiments (Figure 5)

**Day 2**	HCs Mean	Std error	P value vs Cis	
Control	99.2	1.4	0.015	
Cisplatin 30 µM	66.9	9.7	--------	
Cis + Mito 1 µM	78.7	11.2	0.352	
Cis + Mito 2.5 µM	86.5	9.3	0.128	
Cis + Mito 5 µM	66.0	5.8	0.944	
Cis + Mito 10 µM	57.1	19.5	0.441	
Mito 10 µM	99.7	0.2	<0.0001	S
Cis + SQ 50 nM	76.3	6.6	0.459	
Cis + SQ 100 nM	86.5	7.4	0.055	
Cis + SQ 500 nM	88.2	6.0	0.100	
Cis + SQ 800 nM	81.5	19.5	0.255	
SQ 800 nM	98.9	0.p0	<0.0001	S

**Day 3**	HCs Mean	Std error	P value vs Cis	
Control	91.8	3.3	0.015	
Cisplatin 30 µM	59.4	8.5	--------	
Cis + Mito 1 µM	56.5	10.5	0.822	
Cis + Mito 2.5 µM	64.3	3.8	0.702	
Cis + Mito 5 µM	47.0	6.2	0.330	
Cis + Mito 10 µM	48.7	16.3	0.402	
Mito 10 µM	85.4	4.1	<0.0001	S
Cis + SQ 50 nM	68.5	2.5	0.473	
Cis + SQ 100 nM	78.7	7.5	0.134	
Cis + SQ 500 nM	68.7	6.2	0.464	
Cis + SQ 800 nM	57.6	20.2	0.888	
SQ 800 nM	95.9	5.4	<0.0001	S

**Day 4**	HCs Mean	Std error	P value vs Cis	
Control	75.7	10.1	0.018	
Cisplatin 30 µM	39.5	5.3	--------	
Cis + Mito 1 µM	32.8	7.8	0.642	
Cis + Mito 2.5 µM	48.0	8.6	0.563	
Cis + Mito 5 µM	29.6	4.1	0.498	
Cis + Mito 10 µM	29.2	10.4	0.480	
Mito 10 µM	50.7	4.1	<0.0001	S

Cis + SQ 50 nM	57.7	16.4	0.218	
Cis + SQ 100 nM	57.3	14.1	0.230	
Cis + SQ 500 nM	56.8	6.0	0.242	
Cis + SQ 800 nM	38.1	15.4	0.921	
SQ 800 nM	66.4	10.4	<0.0001	S
